# Optimized electrochemical performance of Ni rich LiNi_0.91_Co_0.06_Mn_0.03_O_2_ cathodes for high-energy lithium ion batteries

**DOI:** 10.1038/s41598-019-45531-2

**Published:** 2019-06-20

**Authors:** Seung-Hwan Lee, Seul Lee, Bong-Soo Jin, Hyun-Soo Kim

**Affiliations:** Next-Generation Battery Research Center, Korea Electrotechnology Research Institute, Changwon, 641-120 South Korea

**Keywords:** Batteries, Batteries

## Abstract

We report high electrochemical performances of LiNi_0.91_Co_0.06_Mn_0.03_O_2_ cathode material for high-energy lithium ion batteries. LiNi_0.91_Co_0.06_Mn_0.03_O_2_ is synthesized at various sintering temperatures (640~740 °C). The sintering temperatures affect crystallinity and structural stability, which play an important role in electrochemical performances of LiNi_0.91_Co_0.06_Mn_0.03_O_2_. The electrochemical performances are improved with increasing sintering temperature up to an optimal sintering temperature. The LiNi_0.91_Co_0.06_Mn_0.03_O_2_ sintered at 660 °C shows remarkably excellent performances such as initial discharge capacity of 211.5 mAh/g at 0.1 C, cyclability of 85.3% after 70 cycles at 0.5 C and rate capability of 90.6% at 2 C as compared to 0.5 C. These results validate that LiNi_0.91_Co_0.06_Mn_0.03_O_2_ sintered at 660 °C can be regarded as a next generation cathode.

## Introduction

The importance of energy storage devices is rapidly increasing, and various energy storage devices such as lithium ion batteries (LIBs), sodium-ion battery, electrochemical capacitors (ECs) and hybrid supercapacitors are being studied^1–4^. In the case of ECs, various attempts have been made to improve the energy density; however, it is difficult to realize the high energy density of the LIBs (~200 Wh/kg) which is the greatest advantage for energy storage application^5^. Thus, LIBs are widely used in electric vehicles (EVs), hybrid electric vehicles (HEVs), golf carts, electric bicycles, portable devices and so on.

The energy density of LIBs is mainly determined by the cathode since the commercial carbonaceous anode potential is ~0 V^6^. Among the various cathode candidates, layer structured Li(Ni,Co,Mn)O_2_ (NCM) has been regarded as the most attractive alternative to LIBs owing to relatively modest volume change (LiCoO_2_), high specific capacity (LiNiO_2_) and good thermal stability (LiMn_2_O_4_)^7–9^. Li(Ni_1/3_Co_1/3_Mn_1/3_)O_2_ has been successfully commercialized as a battery cathode, researches on Ni-rich NCM (LiNi_x_Co_y_Mn_1−x−y_O_2_, x > 0.5) have been spotlighted due to its superior capacity (>200 mAh/g, at 4.6 V vs. Li/Li^+^)^10^. Therefore, the future of NCM for high energy LIBs strongly depends on Ni-rich NCM materials.

There are three main reasons for performance degradation of Ni-rich cathode: (i) cation disorder decreases the capacity, closely related to phase transformation of layered structure to a spinel or rock-salt structure. (ii) undesirable materials on the cathode surface from reaction with transition metal ions and electrolyte cause performance decay. (iii) disintegration derived from mechanical which stress deteriorate the long term stability by consuming active lithium^11,12^. It is reported that crystallinity, morphology and structural stability, influenced by the sintering temperatures, all play an important role in the electrochemical performances, especially for Ni-rich cathode^11^. In this paper, we report the synthesis of LiNi_0.91_Co_0.06_Mn_0.03_O_2_ (denoted as NCM91) cathode materials at various sintering temperatures. Also the relationship between the sintering temperatures and the electrochemical performances is investigated.

## Experimental

For higher energy density, spherical precursor NCM91 with a bimodal size distribution was prepared by co-precipitation methodeusing NiSO_4_·6H_2_O, CoSO_4_·7H_2_O and MnSO_4_·H_2_O^12^. Also, NaOH and NH_4_OH solution were also used as a chelating agent. The LiOH·H_2_O was mixed with as-prepared NCM91 in a molar ratio 1.05: 1. The mixture was calcined at 500 °C for 5 h and then sintered at 640~740 °C for 15 h in air. The heating and cooling rates for sintering processes were fixed at 10 °C min^−1^.

The cathodes were prepared using the following process: to fabricate a slurry, active material, conductive carbon black binder (Super P) and polyvinylidene fluoride (PVDF) were mixed in the weight ratio of 96:2:2. *N*-Methyl pyrrolidone (NMP) solvent was then added to form slurry. It was casted on aluminum foil and then dried at 120 °C to remove the NMP solvent and then the aluminum foil was pressed. The 2032 coin cells were assembled with Li metal disc as anode, 1 M LiPF_6_ in ethylene carbonate, dimethyl carbonate, and ethyl methyl carbonate (EC:DMC:EMC 1:1:1, v/v/v/) as electrolyte and polyethylene was used as a separator. All coin cells were assembled in argon-gas-filled glove box.

The structural properties of the NCM91 particles were measured using X-ray diffraction (XRD), field emission scanning electron microscope (FESEM). The electrochemical performances were measured using an electrochemical equipment (TOSCAT-3100, Toyo system). The electrochemical impedance spectroscopy (EIS) was conducted using the electrochemical interface and a frequency response analyzer (Bio-Logic, VSP-300) in the frequency range of 10^−2^ to 10^6^ Hz. The residual lithium (LiOH and Li_2_CO_3_) amounts were obtained via titration method. 2.5 g of NCM91 powder is soaked into 50 mL deionized water. After stirring for 15 min, clear solution (25 mL) is separated by vacuum filtering. While stirring, a flow of 0.1 M HCl is added to the solution and pH is recorded. This experiment is finished when the pH reaches 4.

## Results and Discussion

**Figure 1:**
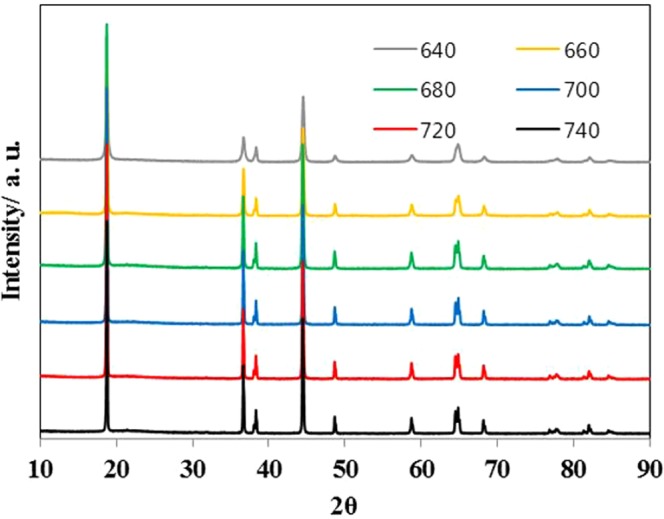
XRD patters of NCM91 sintered at different sintering temperatures.

Figure 1 shows the XRD patterns of NCM91 with different sintering temperatures. All the peaks belong to the hexagonal α-NaFeO_2_ structure (space group *R-3m*) and no impurity peaks are observed. There is no significant difference, except for the diffraction peak intensity, regardless of sintering temperatures. As shown in Fig. S1, the XRD spectrum of the NCM91 sintered at 640 °C shows the broad diffraction peaks and no peak splitting of (006)/(102) and (108)/(110) pairs, resulting from low crystallinity. This is because relatively low sintering temperature is not beneficial to the crystallinity^13,14^. However, NCM91 sintered above 660 °C exhibits sharp diffraction peaks and clear peak splitting, indicating the well-crystallized layered structure. The intensity ratios of (003)/(104) (I(003)/I(104)) is a direct indication of cation mixing, and higher the I(003)/I(104), the lower degree of disorder from Li^+^/Ni^2+^. As shown in Table 1, intensity ratio for as-prepared NCM91 sintered at 640, 660, 680, 700, 720 and 740 °C are 1.07, 1.41, 1.43, 1.41, 1.36 and 1.11, respectively. Zhang *et al*. reported that value less than 1.2 indicates the undesirable cation mixing^15^. Therefore, we can confirm that appropriate sintering temperature could suppress the cation mixing and the NCM91 sintered at 680 °C has the highest I(003)/I(104) value among various sintering temperatures. Although NCM91 sintered at 740 °C, has an identical crystal structure, but also has high Li/Ni cation mixing due to the increase of oxygen vacancy, leading to high Ni^2+^ content^16^. A partial disordering caused by Ni^2+^ migration from transition-metal layer to the lithium layer is expected since the ionic radius of Ni^2+^ (0.69 Å) is similar to that of Li^+^ (0.76 Å).

**Table 1:** I(003)/I(104) ratios of NCM91 sintered at different sintering temperatures.

Sintering temperature (^o^C)	740	720	700	680	660	640
I(003)/I(104)	1.11	1.36	1.41	1.43	1.41	1.07

The microstructure of NCM91 sintered at temperatures from 640 to 740 °C are shown in Fig. S2. All sample exhibit the spherical granule shape composed of numerous primary particles. The primary particle size increases with agglomeration as the sintering temperature is increased. As shown in Fig. S3, the average particle sizes of samples sintered at 660 and 740 °C are 0.34 and 0.70 µm, respectively. It can be explained by favorable growth kinetics at high sintering temperature. Although the primary particle size increases due to high sintering temperature, the size of spherical granule is maintained well. It can be elucidated that Ni_0.91_Co_0.06_Mn_0.03_(OH)_2_ precursor serve as a core and LiOH·H_2_O serve as the “nutrient”, diffused into the precursor during the synthesis process^13^. It can be inferred that structural and morphological characterization will affect the electrochemical performances of NCM91.

**Figure 2:**
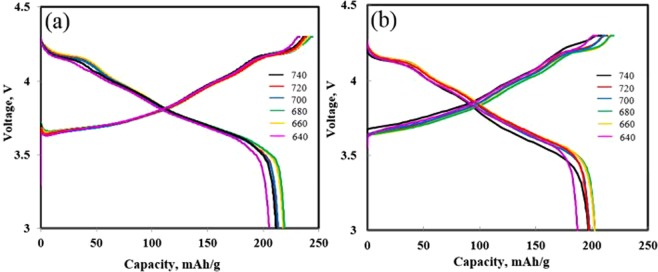
Charge-discharge profile evolutions of NCM91 at (**a**) 0.1 C and (**b**) 0.5 C.

The electrochemical performances of the NCM91 samples which were prepared in thick electrode laminates with high mass loading per area (approximately 14.7 mg/cm^2^), since the high areal capacity is necessary for practical application. Figure 2 presents the charge-discharge profiles of (a) 0.1 C and (b) 0.5 C for NCM91 sintered at various sintering temperatures. We can confirm that the capacity of all samples is not significantly different. Among them, the sample sintered at 680 °C shows the highest capacity due to fast electrode kinetics and high material utilization^17^. It can be explained by high cationic ordering and particle size. During both cycles, the sample sintered at 680 °C delivers 213.4 and 202.4 mAh/g at 0.1 C and 0.5 C, respectively. The capacity is proportional to the sintering temperature up to 680 °C. However, the excessive sintering temperatures above 680 °C cause the replacement of Li^+^ site by Ni^2+^ due to the presence of a large amount of oxygen deficiency, as shown in Table 1. The surface oxygen deficiency, related to the structural degradation and interface reaction at the cathode and electrolyte interface, is the one of the most important factor which declines the electrochemical performance^18^.

**Figure 3:**
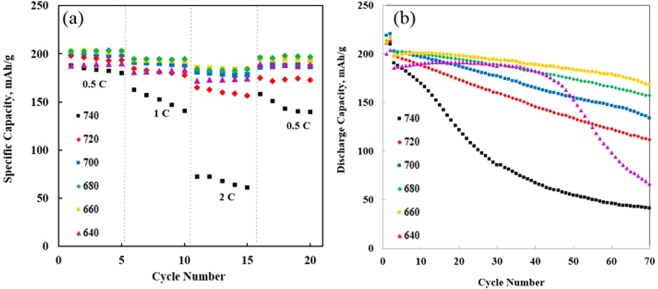
(**a**) Rate performance and (**b**) cycling performance of NCM91 sintered at different sintering temperatures.

In the rate performance test (Fig. 3(a)), the capacity retentions of the all cells decay in the 15 cycles from 0.5 to 2 C charge-discharge rate. The NCM91 are cycled at each rate for 5 cycles. Although the retention differences of all cells are small at a low C-rate, it is evident that the retention difference increased between cells as the C-rate increased. The NCM91 sintered at 740 °C shows the worst rate capability compared to the other sintering temperatures in the entire C-rate range, suggesting excessive sintering temperature leads to sluggish kinetics of lithium ion diffusion, originated from high internal resistance. On the other hand, the capacity retention of the sample sintered at 660 °C and 680 °C have superior retentions during the 15 cycles and the retentions are 97.5% (196.5 mAh/g) and 96.1% (194.6 mAh/g) at 2.0 C rate, respectively. Moreover, they can recover nearly the same initial capacity when the C-rate is decreased back to 0.5 C. It implies that optimized sintering temperature can synthesize the highly crystallized layered structure with enough space for fast lithium-ion intercalation.

Figure 3(b) shows the long-term cyclability of NCM91 at 0.5 C in the voltage rage between 3.0 and 4.3 V. Within 70 cycles, no obvious capacity fading was observed for NCM91 sintered at 660 °C, however, the retention of NCM91 sintered above 680 °C decreases steadily from the beginning. The sample sintered at 640 °C and 740 °C show slightly low discharge capacities compared to others. It can be explained by unoptimized sintering temperature which induced structural weakness such as poor crystallinity (640 °C), serious cation mixing (640 and 740 °C) and long lithium ion movement pathways (740 °C)^19^. Although the sample sintered at 680 °C delivers the highest discharge capacity of 202.4 mAh/g in the first cycle, its low retention makes it less attractive. The sample sintered at 660 °C delivers a relatively low capacity of 197.3 mAh/g, it has an excellent retention of 85.3% under the same condition, implying that it has enough active sites for smooth and fast lithium ion diffusion with enhanced interfacial stability^20^. Ni-rich cathode is structurally unstable owing to oxygen release at the surface and structure changes since Ni-rich cathode is inevitable to lose oxygen atoms and then generate oxygen defects because of low bonding energy between Ni^3+^ and O^2−^. Moreover, oxygen and Ni^4+^ ion can lead to decomposition of the electrolyte. These phenomenon results in serious performance decay and safety problems^21^.

**Figure 4:**
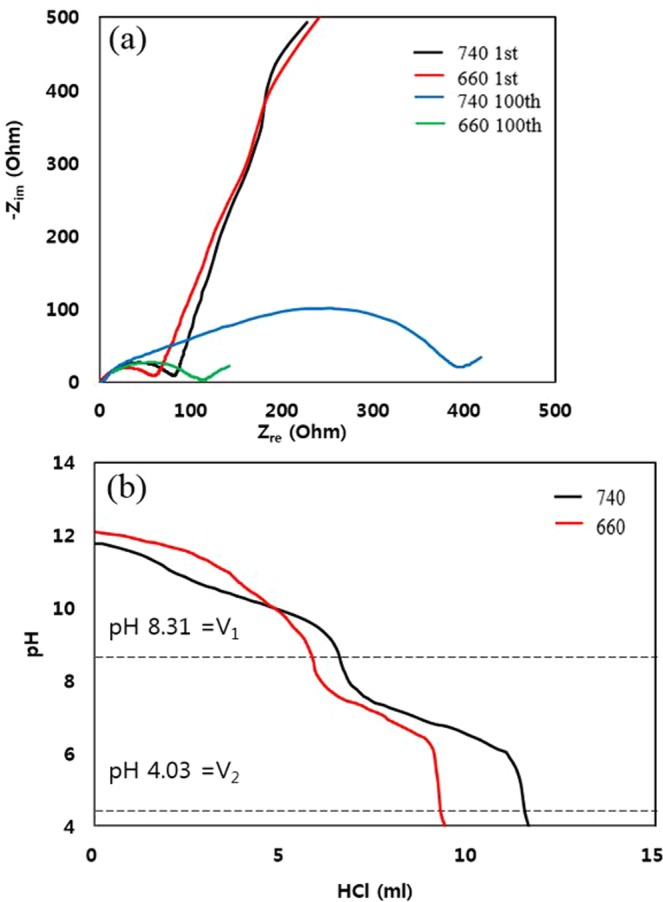
(**a**) Nyquist plots and HCl-titration graph of NCM91 sintered at 660 and 740 °C.

EIS was performed to investigate the enhanced cycle performance of the NCM 91 sintered at 660 °C. The Nyquist plots in Fig. 4(a) consists of three components: electrolyte resistance (R_s_) in the high frequency, the charge transfer resistance (R_ct_) in the medium frequency and the Warburg impedance in the low frequency^22^. The values of electrolyte resistance (R_s_) were almost same since NCM91 use the same electrolyte and the difference in R_ct_ is small after first cycle. On the other hand, there is a big difference in R_ct_ value after cycle test. The R_ct_ of NCM91 sintered 740 °C was significantly increased from 78.4 to 399.2 Ω after 70 cycles, while R_ct_ of NCM91 sintered 660 °C is efficiently suppressed by highly-crystallized structure with abundant space for smooth lithium ion diffusion. The R_ct_ value of the sample sintered at 660 °C is about one fourth compared to the sample sintered at 740 °C. It demonstrates that the more effective charge-carrier transport occurs at the electrode and electrolyte interfaces. Therefore, we can conclude that lower R_ct_ can be regarded as an important factor for capacity fading since the higher R_ct_ enhanced the kinetic barrier for lithium ion diffusion, resulting in rapid capacity decay upon cycling^23^. These are in line with rate capability and cyclability, as mentioned in Fig. 3(a,b), respectively. Figure 4(b) shows the HCl-titration curves of NCM91 sintered at 660 and 740 °C. The unwanted materials caused by interfacial side reaction is contributed mainly from two sources: (i) residual lithium such as Li_2_CO_3_ and LiOH in the NCM91, resulting from moisture absorption and reduction of Ni^3+^ to Ni^2+^^24^ (ii) hydrogen-containing compounds derived from ion exchange at the surface of NCM91 (H^+^ (water) ↔ Li^+^ (surface or outer bulk)). However, the latter is generally negligible^25^. The amount of HCl used in the HCl titration up to pH 4 of sample sintered at 660 °C is less than that of 740 °C. It indicates that LiOH and Li_2_CO_3_ exist at the NCM91 surface and appropriate sintering temperature can suppress the residual lithium. The amount of the LiOH and Li_2_CO_3_ is calculated (Table 2) and could be obtained by the following equations^26,27^:

1 $${\rm{LiOH}}+{\rm{HCl}}\to {\rm{LiCl}}+{{\rm{H}}}_{{\rm{2}}}{\rm{O}}$$

2 $${{\rm{Li}}}_{{\rm{2}}}{{\rm{CO}}}_{{\rm{3}}}+{\rm{2HCl}}\to {\rm{2LiCl}}+{{\rm{H}}}_{{\rm{2}}}{\rm{O}}+{{\rm{CO}}}_{{\rm{2}}}$$

**Table 2:** The variation of LiOH and Li_2_CO_3_ contents of NCM91 sintered at 660 and 740 °C.

Residual lithium	LiOH (wt%)	Li_2_CO_3_ (wt%)
740 °C	0.94	3.94
660 °C	0.66	3.49

The total amount of residual lithium of sample sintered at 660 °C is less than that of 740 °C, which alleviates the swelling phenomenon and suppress the performance degradation^28^. It is closely related to the gelation of the NCM91 slurry due to the increased pH^29^. Therefore, we can conclude that the total amount of residual lithium is reduced by optimizing sintering temperature.

**Figure 5:**
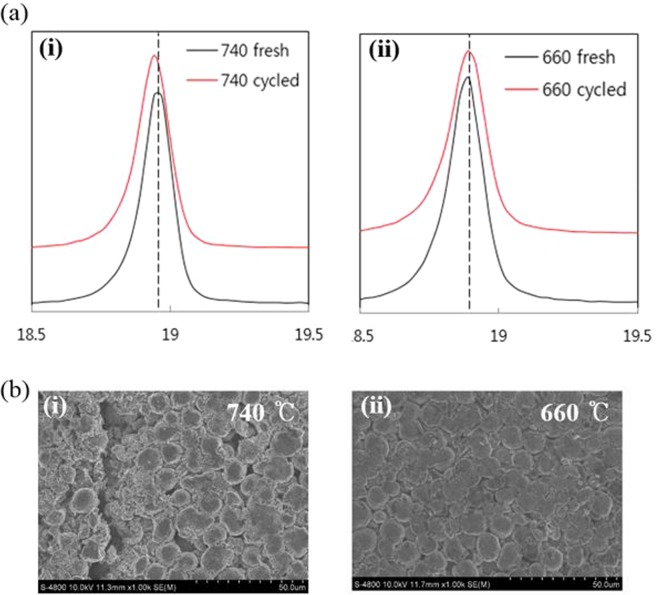
(**a**) XRD patterns and (**b**) SEM image of NCM91 sheet after cycle test.

Figure 5 shows the (a) XRD pattern and (b) SEM images of the NCM91 sintered at 660 °C and 740 °C after cycle test for further insight into the effect of sintering temperature. We can confirm that the peak position of the sample sintered at 660 °C presents the almost identical while slight peak shift to lower angles is observed for sample sintered at 740 °C. It can be explained by two reasons: (i) the continuously expansion of lattice during cycling causes the breakdown of the original NCM91 structure; (ii) the brokenness of particle causes the broaden peak^5^. From SEM image, we can confirm that the sample sintered at 660 °C shows smooth surface without evident cracks and mitigated structural degradation owing to buffer space, NCM91 and electrode structure integrity. The minor crack can be explained by rolling process^30^. However, the sample sintered at 740 °C has significant crack on the surface under the same condition. As shown in Fig. S3(c,d), the surfaces of NCM91 were stable without cracks regardless of sintering temperature before cycling. It is associated with the primary particle size inside the spherical-granule NCM91. The NCM91 sintered at 660 °C have relatively smaller sized particles than that of 740 °C, as shown in Fig. S3. Within the densely packed secondary particles, the crack formation is caused by stress, resulting from the anisotropic lattice expansion and contraction of grains during charge-discharge process and then strain at the grain boundaries is generated^31,32^. The crack can lead to (i) poor grain-to grain connections, resulting in inferior electrical conductivity and even loss of active materials due to fragmentation; (ii) phase transformation, corrosion and side reaction via creation of fresh surface that will react with electrolyte. All these lead to rapid buildup of electrode resistance and quick capacity fading. Therefore, crack can be considered to be one of the most important factor for performance degradation^33^.

## Conclusions

We synthesized layered NCM91 cathode with different sintering temperature from 640 to 740 °C for the purpose of achieving superior electrochemical performances. The effects of the sintering temperatures on the structural properties and electrochemical performances of NCM91 are investigated. The results indicate the importance of optimum sintering temperature and the NCM91 sintered at 660 °C delivers excellent discharge capacity, rate performance, cycle performance, benefiting from good crystallinity, low cation mixing and small primary particle size. Therefore, we can conclude that the electrochemical performances of NCM91 is sensitive to the sintering temperature and optimized sintering temperature of NCM91 was found to be around 660 °C. The findings indicate that NCM91 sintered at 660 °C can be regarded as a promising cathode for the next-generation lithium ion batteries.

## Supplementary information

Supporting Information
