# Supplementary material for: Optimized electrochemical performance of Ni rich LiNi0.91Co0.06Mn0.03O2 cathodes for high-energy lithium ion batteries
Source: Sci Rep. 2019 Jun 20;9:8901. doi: 10.1038/s41598-019-45531-2 (PMC6586611; doi:10.1038/s41598-019-45531-2)
Supplement: Supplementary file 1 — Supporting Information [file 41598_2019_45531_MOESM1_ESM.doc]

Supporting Information

Optimized electrochemical performance of Ni rich LiNi_0.91_Co_0.06_Mn_0.03_O_2_ cathodes for high-energy lithium ion batteries

**Seung-Hwan Lee^a^, Seul Lee^a^, Bong-Soo Jin^a^, Hyun-Soo Kim^a,*^**

*^a^Next-Generation Battery Research Center, Korea Electrotechnology Research Institute, Changwon 641-120, South Korea*

Author(s) e-mail addresses

^*^ hskim@keri.re.kr


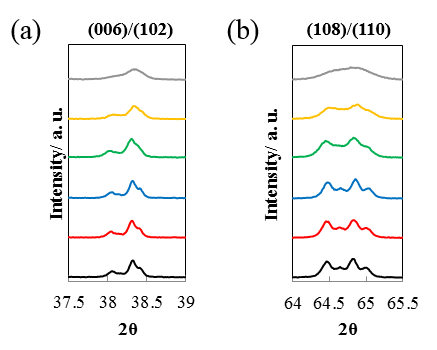


**Fig. S1†**. Enlarged XRD patterns of from (a) 37.5 - 39.0º and (c) 64.0 - 65.5º of NCM91 with different sintering temperatures.


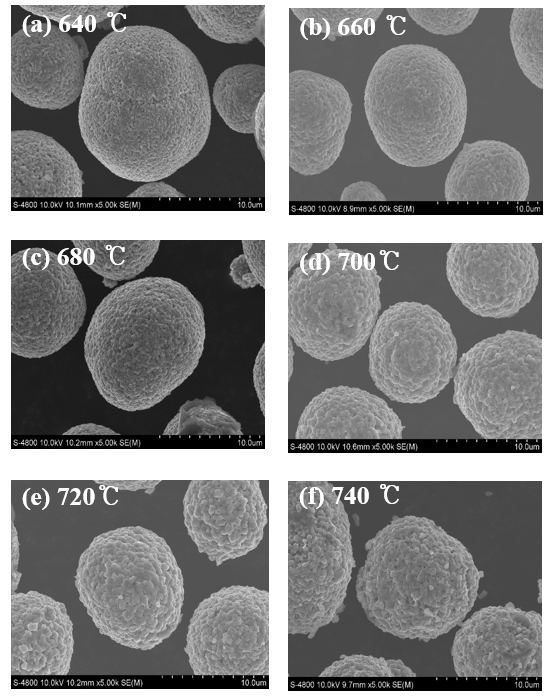


:

**Fig. S2†**. SEM image NCM91 with different sintering temperatures: (a) 640 ^o^C (b) 660 ^o^C (c) 680 ^o^C (d) 700 ^o^C (e) 720 ^o^C and (f) 740 ^o^C.


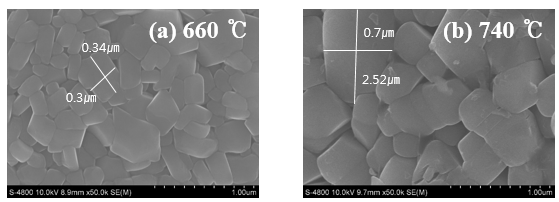


**
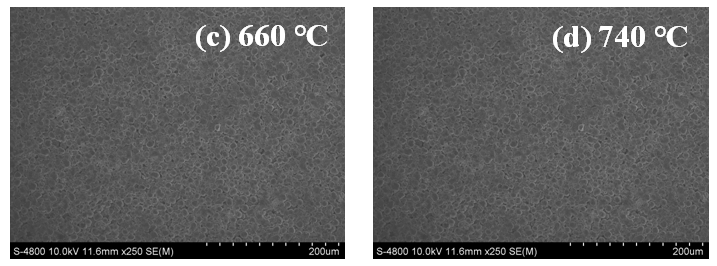
**

**Fig. S3†**. Enlarged SEM image NCM91 sintered at (a) 680 ^o^C and (b) 740 ^o^C. (c) SEM image of NCM91 sheet sintered at 660 ^o^C and 740 ^o^C after cycle test.
